# Clinical Implications of DNA Repair Defects in High-Grade Serous Ovarian Carcinomas

**DOI:** 10.3390/cancers12051315

**Published:** 2020-05-21

**Authors:** Michela Camilla Milanesio, Silvia Giordano, Giorgio Valabrega

**Affiliations:** 1 Department of Oncology, University of Torino, Candiolo, 10060 Torino, Italy; michela.milanesio@unito.it (M.C.M.); silvia.giordano@unito.it (S.G.); 2 Candiolo Cancer Institute, FPO-IRCCS, Strada Provinciale 142 Km 3.95, Candiolo, 10060 Torino, Italy

**Keywords:** epithelial ovarian cancer, DNA repair deficiency, DNA homologous recombination, DNA mismatch repair, PARP inhibitors, BRCA reversion mutations

## Abstract

Despite significant improvements in surgical and medical management, high grade serous ovarian cancer (HGSOC) still represents the deadliest gynecologic malignancy and the fifth most frequent cause of cancer-related mortality in women in the USA. Since DNA repair alterations are regarded as the “the Achille’s heel” of HGSOC, both DNA homologous recombination and DNA mismatch repair deficiencies have been explored and targeted in epithelial ovarian cancers in the latest years. In this review, we aim at focusing on the therapeutic issues deriving from a faulty DNA repair machinery in epithelial ovarian cancers, starting from existing and well-established treatments and investigating new therapeutic approaches which could possibly improve ovarian cancer patients’ survival outcomes in the near future. In particular, we concentrate on the role of both Poly (ADP-ribose) Polymerase (PARP) inhibitors (PARPis) and immune checkpoint inhibitors in HGSOC, highlighting their activity in relation to BRCA1/2 mutational status and homologous recombination deficiency (HRD). We investigate the biological rationale supporting their use in the clinical setting, pointing at tracking their route from the laboratory bench to the patient’s bedside. Finally, we deal with the onset of mechanisms of primary and acquired resistance to PARPis, reporting the pioneering strategies aimed at converting homologous-recombination (HR) proficient tumors into homologous recombination (HR)-deficient HGSOC.

## 1. DNA Repair Defects: the “Achille’s heel” of High-Grade Serous Ovarian Cancers

High-grade serous epithelial ovarian cancer (HGSOC) represents the deadliest malignancy among gynecologic cancers and achieves the fifth rank among the most frequent causes of cancer-related mortality in women in the United States [1]. Advanced disease is diagnosed in nearly 75% of epithelial ovarian cancer (EOC) patients and this finding is primarily responsible for the modest 5-year overall survival (OS) rate uncovered in women harboring EOC [2]. Primary surgical cytoreduction followed by platinum-based chemotherapy constitutes the therapeutic strategy backbone in ovarian carcinomas, but, despite important improvements attained in medical treatments by the addition of taxanes, pegylated liposomal Doxorubicin, and Bevacizumab, patient survival has not changed noticeably, suggesting that alternative approaches are needed [2]. Indeed, despite initial response to platinum-based chemotherapy, most patients with this type of cancer experience disease relapse and ultimately develop platinum resistance. This is strictly related both to extensive intratumoral heterogeneity in primary high-grade serous carcinomas (accounting for about 70% of EOCs) and spatial and temporal genomic evolution under the selective pressure of medical treatments [3,4,5].

The prerogative for cell survival relies on the ability of maintaining genomic stability, a task involving the regulation of DNA replication, DNA repair and cell-cycle progression, carried out by the coordinated action of the DNA damage response (DDR) machinery. The DDR machinery is meant to deal with the two main types of DNA lesions, i.e., single-strand breaks (SSBs) and double-strand breaks (DSBs); whether DNA is not repaired, replication stress rises. While, on one hand, a defective DDR may lead to neoplastic transformation and proliferation by increasing cell mutational load, on the other hand, it can be exploited for therapeutic purposes as cells harboring a specific DDR defect can become reliant on other targetable repair pathways for perpetuating their survival [6,7]. In particular, since evidence from The Cancer Genome Atlas Research Project indicates that abnormalities of genes and pathways involved in DNA damage repair contribute to impairment of homologous recombination (HR) in almost 50% of HGSOC, DNA repair defects are regarded as the “Achille’s heel” of this group of malignancies [1,6]. Moreover, pathways other than the homologous recombination system could be implicated in HGSOC genetic instability (i.e., DNA mismatch repair) [8,9,10].

## 2. Homologous Recombination Deficiency in EOC

### 2.1. Relevant Proteins and Pathways

Homologous recombination preserves cell genetic information by fixing DNA DSBs in a highly reliable fashion. In order to minimize the accumulation of DNA alterations deriving from both direct DNA damage and inaccurate repair systems, the HR pathway coordinates DNA DSBs mending by employing a sister chromatid as DNA template for nucleotide sequence regeneration [11,12].

**Figure 1:**
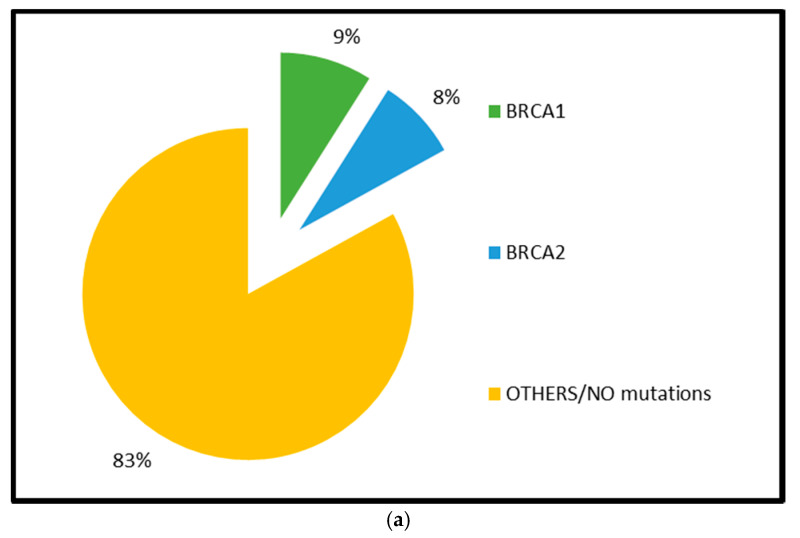

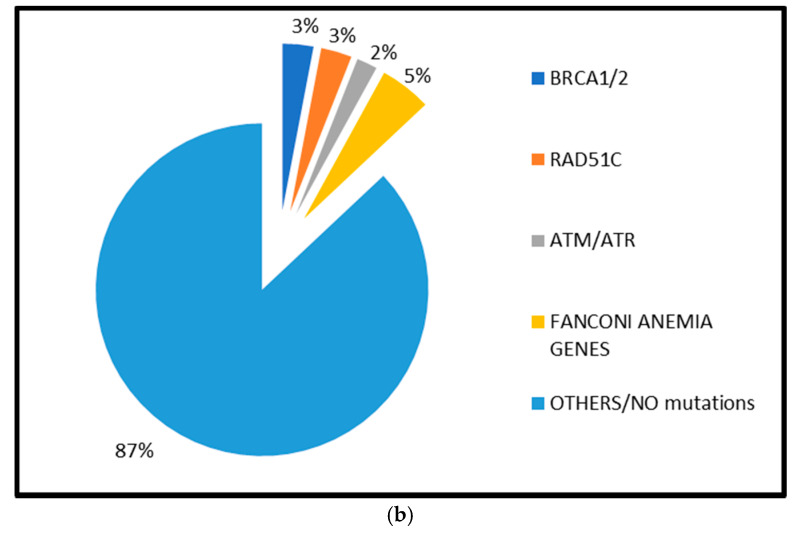
Distribution of homologous recombination deficiency (HRD) genes’ mutations in high grade serous ovarian cancers (HGSOCs): (**a**) germline mutations; (**b**) somatic mutations (data from The Cancer Genome Atlas [1]).

As far as homologous recombination in epithelial ovarian cancer is concerned, both germline and somatic BRCA1/2 mutations constitute the most common alterations, being detected in roughly 17% and 3% of HGSOCs, respectively [1]. BRCA1 promoter hypermethylation-induced epigenetic silencing is numbered among other mechanisms known to confer HR deficiency in EOC and is reported in approximately 10% to 20% of HGSOCs; interestingly, this epigenetic modification occurs exclusively for BRCA1 gene (not for BRCA2) and is mutually exclusive of BRCA1/2 mutations, suggesting a strong selective pressure to inactivate BRCA via either mutation or epigenetic silencing in ovarian cancer [1]. Other alterations responsible for the disruption of the HR machinery integrity in EOC include mutations in several Fanconi Anemia genes (mainly PALB2, FANCA, FANCI, FANCL, and FANCC), in core HR RAD genes (such as RAD50, RAD51, RAD51C, and RAD54L) and in DNA damage response genes involved in HR (such as ATM, ATR, CHEK1, and CHEK2) (Figure 1) [1,13,14,15,16].

#### 2.1.1. BRCA1 and BRCA2 Mutations

BRCA1 and BRCA2 genes encode protein products which participate together with other tumor suppressor proteins in the formation of complexes committed to chromosome damage repair by HR [17]. Interestingly, a distinctive clinical phenotype has been reported in association with HR-deficient cancers, especially those characterized by BRCA1/2 mutations. Particularly, women harboring BRCA1/2-mutated ovarian cancers exhibit both enhanced response to platinum-based chemotherapeutic regimens and significantly improved OS when compared with patients diagnosed with non-BRCA-mutated tumors; captivatingly, these effects are more prominent for BRCA2 mutation carriers, who show even longer survival with respect to BRCA1 patients [18,19,20]. Conversely, less favorable outcomes have been detected in EOCs displaying BRCA1 promoter hypermethylation-induced epigenetic silencing, suggesting that different mechanisms of HR deficiency may underlie distinct clinical phenotypes. Even though the better prognosis accompanying BRCA-mutated cancers may be mainly explained by the higher rates of responsiveness to platinum-based chemotherapy, a more indolent natural history due to intrinsic biologic peculiarities may also play a role [1,18,21]. Several lines of evidence indicate that BRCA1/2-mutated tumors may harbor an increased mutational load which renders them more immunogenic when compared with their HR-proficient counterpart [19,22,23,24,25]. Finally, in terms of pattern of recurrence, BRCA1/2-mutated tumors are more prone to spread to visceral organs (giving rise to liver, lung, adrenal, spleen and brain metastases), this characteristic being more pronounced for BRCA1- mutated tumors [20].

#### 2.1.2. Role of Poly (ADP-ribose) Polymerase (PARP) in HR deficiency

In targeting HR-deficient ovarian cancers different strategies can be adopted, ranging from conventional chemotherapy (platinum analogues, pegylated liposomal Doxorubicin, Topotecan, Etoposide and Gemcitabine) to PARP and cell-cycle/DNA-damage checkpoint inhibitors [6,26,27].

The PARP family of enzymes encompasses 17 members which are involved in virtually every essential pathway of cellular biology due to their role in ADP-ribose post-translational modification of proteins using NAD^+^. Specifically, PARP1, PARP2, and PARP3 are mainly implicated in the DNA damage response, since they are committed to identify DNA alterations and coordinate repair factors assembly to the DNA break sites [28].

**Figure 2:**
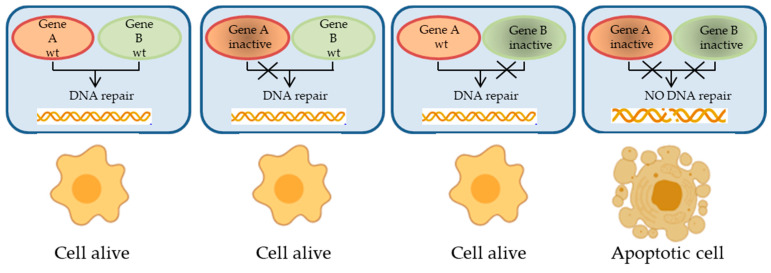
Schematic illustration of synthetic lethality. The concomitant alteration of two genes (defined as A and B), generally involved in complementary pathways, leads to cell death, while loss of function of only one of them does not. Synthetic lethality exploits the notion that the presence of a mutation in a cancer gene is often associated with a new vulnerability that can be targeted therapeutically.

The concept of synthetic lethality (defined as a genetic combination of mutations in two or more genes that leads to cell death, whereas a mutation in only one of the genes does not) (Figure 2) in the DNA damage response has recently had a translational application with the finding that PARP inhibitors are toxic to HR-defective cells. Nevertheless, the exact biological bases underlying the synthetic lethal interaction existing between PARPis and HR disruption have not been completely elucidated yet. Originally, PARP1 was pointed at as an essential effector taking part in DNA base excision repair (BER), thus preventing DNA SSBs from converting into more cytotoxic DSBs, routinely repaired by the HR machinery. In this scenario, PARPis can lead to a higher rate of DNA DSBs that remain unrepaired in HR-deficient cells, subsequently causing cytotoxic death [6,7]. However, recent observations recognize a role for PARP1 also in the so-called “error-prone alt-EJ or microhomology-mediated end joining” (MMEJ) repair pathway of DNA DSBs, underscoring how HR-deficient ovarian and breast tumors display a compensatory increase in the Polθ/PARP1-mediated alt-EJ pathway that is strictly involved in their survival and proliferation [29,30,31]. Therefore, this finding may disclose a possible synthetic lethal combination between HR deficiency and inhibition of the Polθ/PARP1 axis. In addition, PARPis are thought to be toxic to BRCA 1/2-mutant cancers by their ability to trap PARP1 enzyme on DNA. Indeed, the most effective PARPis are those that most successfully trap PARP proteins on DNA, generating a bulky protein-DNA adduct which leads BRCA 1/2-mutated cancer cells to death [32,33].

#### 2.1.3. Focus on p53 Protein

HGSOC is one of the tumor types presenting the highest prevalence of p53 mutations (around 96% of cases), suggesting that p53 is a critical tumor suppressor for ovarian cancer [1]. As p53 mutations are already found at the stage of early serous tubal intraepithelial lesions, it is believed that p53 alteration is an early event in the evolution of HGSOC [34]. Anticancer agents induce apoptosis in ovarian cancer cells by damaging DNA in dividing cells. While normal cells respond to such stress conditions by increasing p53 expression, in the case of p53 mutation/absence, the cell is unable to initiate DNA damage-induced apoptosis or to enter cell cycle arrest, thus undergoing continuous proliferation [35]. Unfortunately, there are no currently approved drugs able to reactivate p53.

### 2.2. Therapeutic Implications

While PARP inhibitors efficacy in treating BRCA1/2-mutated HGSOCs has been firmly established [36], the clinical benefit demonstrated by PARPis in BRCA wild-type (BRCA WT) patients has been rising awareness about the “beyond BRCA” efficacy of these drugs (Table 1).

In this landscape, the Study 19 [37], the ARIEL3 [38], the ENGOT-OV16/NOVA [39], and the QUADRA [40] clinical trials indirectly laid the groundwork for the scientific rationale supporting the disruption of the DNA-damage checkpoint system in BRCA WT HGSOC.

Assessing the efficacy of the oral PARP inhibitor Olaparib as maintenance monotherapy in women with platinum-sensitive, relapsed HGSOC experiencing a partial or complete response to their last platinum-based chemotherapy, Study 19 was the first randomized phase 2 clinical trial to demonstrate that patients with BRCA1 or BRCA2-mutated HGSOCs are likely to derive the greatest clinical benefit from treatment with a PARP inhibitor [37,41]. Interestingly, data from a retrospective analysis conducted by stratifying patients according to their BRCA mutational status demonstrated that patients harboring a BRCA-mutated HGSOC showed a significantly longer median progression-free survival (PFS) in the Olaparib group than in the placebo group and the extent of this PFS improvement substantially outperformed results formerly detected in the overall population and in the BRCA WT subgroup. Nonetheless, despite this strong evidence underpinning the biological rationale of synthetic lethality between HR deficiency deriving from BRCA mutations and PARP inhibitors, a significant PFS improvement was also highlighted in women with BRCA WT ovarian carcinomas randomly assigned to the Olaparib arm versus placebo. This observation might be likely due to the heterogeneous framework of the BRCA WT population in Study 19, since it likely encompassed patients whose ovarian cancers harbored mutations in other-than-BRCA genes strictly involved in the DNA homologous recombination.

The ARIEL3 and the ENGOT-OV16/NOVA randomized phase 3 clinical trials further assessed the effect of mutations in other-than-BRCA HRD genes on response to PARP inhibitors in HGSOCs. Indeed, these two interventional studies respectively demonstrated that maintenance therapy with Rucaparib and Niraparib (both oral PARPis) in women with platinum-sensitive, recurrent HGSOC undergoing a partial or complete response after their last platinum-based chemotherapy prolonged PFS not only in BRCA-mutated tumors but also in HRD-positive BRCA-WT HGSOCs. When compared with placebo in nested patient subpopulation analyses, both Rucaparib and Niraparib showed a declining trend of efficacy in terms of improved PFS: the most appealing results were achieved by patients with BRCA-mutant carcinomas, with a gradually weakening effect in the HRD-positive and the intention-to-treat populations. Despite this unequivocal tendency, focusing on BRCA-WT HGSOCs harboring other-than-BRCA mutations impairing the DNA homologous recombination system, the aforementioned PARP inhibitors significantly lowered the risk for disease progression with respect to placebo, highlighting how the HRD status may provide predictive information about the potential treatment benefit deriving from PARPis-mediated synthetic lethality [38,39,42].

Finally, the QUADRA phase 2 clinical study explored the activity of Niraparib monotherapy in heavily pre-treated ovarian cancer patients, including women harboring primary or acquired platinum-resistant or platinum-refractory HGSOC, BRCA-mutated and BRCA WT, and HRD-positive and HRD-negative carcinomas. Consistent with the ENGOT-OV16/NOVA trial results, the QUADRA study highlighted a continuum of clinical benefit with late-line Niraparib monotherapy in subsets of patients identified by clinical and molecular biomarkers. Significantly, a noteworthy activity of Niraparib was reported in women with platinum-sensitive ovarian cancer characterized by homologous recombination deficiency, a patient subgroup which gathered together both BRCA-mutated and BRCA WT/HRD-positive HGSOC [40,43].

Recently, the 2019 European Society for Medical Oncology (ESMO) Congress set a ground-breaking turning point in the forthcoming management of ovarian cancer treatment in the first-line setting. The innovative leitmotif of the majority of research clinical studies lies in the attempt to demonstrate the efficacy of early introduction of PARP inhibitors in the treatment algorithm of advanced OCs, regardless of their BRCA mutational status. This approach aims at dealing with a scenario where available therapies and active clinical surveillance do not address the high unmet need for 85% of OC patients who are going to experience disease recurrence or progression after standard-of-care (SOC), platinum-based chemotherapy for their advanced carcinomas. The PRIMA/ENGOT-ov26-GOG-3012 [44], the PAOLA-1/ENGOT-ov25 [45], and the VELIA/GOG-3005 [46] clinical trials demonstrated the efficacy of PARPis in newly-diagnosed HGSOCs, highlighting a significantly longer PFS in the intention-to-treat population, including both BRCA-mutated and BRCA wild-type ovarian cancers.

Currently, two commercial genomic scar assays have been tested in clinical trials to identify tumors with HRD in ovarian cancer. The first one is “myChoice HRD” assay by Myriad, which detects loss of heterozygosity (LOH), telomeric allelic imbalance (TAI) and large-scale state transitions (LST) across the genome. The output of this assay is presented as “HRD score”: a tumor with an HRD score ≥ 42 is labeled as HRD-positive. This test is used as the diagnostic companion of Niraparib trials. The “FoundationFocus CDx BRCA LOH” is designed to detect the presence of mutations in the BRCA1/2 genes and the percentage of the genome affected by LOH in DNA from tumor tissue samples of patients with ovarian cancer. According to the FoundationFocus test, tumors are categorized as LOH-high if the score is ≥ 16. This test is used as the diagnostic companion of Rucaparib trials. Unfortunately, it is unknown whether these two tests are also predictive of benefit from PARP inhibitors different from the ones they were developed for and, most importantly, it is unclear why subsets of HRD negative patients benefit from PARP inhibitors both in first line (Niraparib) and at relapse (Niraparib and Rucaparib). For these reasons, the current position of ASCO (American Society of Clinical Oncology) is to not recommend routine tumor testing using currently available homologous recombination deficiency (HRD) assays [47].

More academic work is therefore needed to bring to the clinics a reliable test that could predict benefit from most if not all PARPis.

### 2.3. Immunologic Features and Related Clinical Implications in HR-Deficient EOC

Cancer cells are able to escape immune-mediated killing by triggering different immune checkpoint pathways which lead to an immunosuppressive cancer “habitat”. Monoclonal antibodies directed against cancer immune response inhibitory actors are able to boost immune anti-tumor reaction, enhancing elimination of tumor cells by the immune system [48].

Hypermutated cancers, such as melanomas and lung carcinomas, are extremely susceptible to immune checkpoint inhibitors (ICIs) (i.e., anti-CTLA-4 (cytotoxic T-lymphocyte antigen-4), anti-PD-1 (programmed death-1) and anti-PD-L1 (programmed death-1 ligand) antibodies) [49,50]. The enhanced activity of ICIs in hypermutated lesions seems to rely on the higher rate of tumor-specific neoantigens which peculiarly characterizes these cancers; indeed, while the generation of a great multiplicity of neoantigens attracts a pronounced number of tumor-infiltrating lymphocytes (TILs) within tumor tissues, T cell antitumoral cytotoxic activity is negatively counterbalanced by the overexpression of immune checkpoints, such as PD-1 or PD-L1 [50].

Despite their relatively low mutational load, EOCs do not have to be considered as “non-immunogenic” human cancers. Several lines of evidence underscore how EOCs could elicit a detectable reaction sustained by the host immune system in terms of tumor-specific lymphocytes and antibodies [51,52]. Bobisse et. al. [53] identified CD8+ T cells recognizing specific tumor neo-epitopes in nearly 90% of patients with immunotherapy-naïve, heavily pre-treated recurrent advanced EOCs: CD8+ lymphocytes were detected both within tumor tissue (i.e., TILs) and in peripheral blood, with discordant neoantigen specificity and avidity among these two immune cell populations; of note, gene signatures of cancers in which hosts displayed neoantigen-specific peripheral blood T lymphocytes were enriched for genes involved in antigen-processing/presentation pathways and PD-1 signaling. Furthermore, identification of neo-epitopes by circulating T cells was significantly enhanced in patients harboring BRCA1/2-mutated ovarian carcinomas.

Due to the defective activity of their HR DNA repair machinery, BRCA1/2-mutated HGSOCs exhibit a higher tumor mutational burden, thus expressing more tumor-specific neoantigens and harboring increased amounts of TILs and PD-1/PD-L1 [23]. This evidence supports the greater immunogenicity typifying HR-deficient EOCs with respect to their HR-proficient counterpart [23] and, in order to clarify this aspect, recent studies have been investigating the association and prognostic significance of BRCA1/2 mutational status with neoantigen load, number of TILs, and expression of PD-1/PD-L1 in HGSOC.

In this scenario, Strickland et al. [23] proved significantly higher predicted neoantigens, TILs density and PD-1/PDL-1 expression in BRCA1/2-mutated ovarian tumors when compared to cancers without alterations in homologous recombination genes. Of note, improved overall survival was significantly and independently associated with both BRCA1/2 mutational status and number of TILs, underpinning a strong correlation between BRCA1/2-mutation status, immunogenicity and survival in HGSOCs [23]. Taken together, these findings seem to forecast a potentially enhanced susceptibility of BRCA1/2-mutated HGSOCs to PD-1/PD-L1 inhibitors with respect to HR-proficient cancers.

Despite this evidence, phase 1b and 2 clinical trials aiming at assessing the activity of single-agent anti-PD-1 or anti-PDL-1 therapy in recurrent EOC attained modest results, with objective response rates ranging from 8% to 10% and median PFS achieving 8-week duration [54,55]. Starting from this background, researchers have tried to uncover the mechanisms underlying immune resistance in EOC. Odunsi pointed at the profoundly immunosuppressive microenvironment characterizing EOC as the primary obstacle to immunotherapy efficacy, highlighting the potential synergism between immunotherapeutic compounds and conventional chemotherapy/targeted agents as a promising mean to overcome immune resistance [52]. This perspective is supported by the evidence that an efficient immune response is based on a well-defined orchestration of different and interrelated aspects, such as production of effective numbers of cytotoxic T cells with high avidity for tumor antigens, infiltration of CD8+ T cells into the tumor, detection of tumor antigens, and, finally, enhancement of an immune antitumor response [56]. In this landscape, combining immune checkpoint inhibitors with standard chemotherapy drugs and/or target agents could simultaneously act on different steps of immune response activation and elicitation, potentially dribbling and weakening resistance mechanisms [56,57]. Particularly, the association of PARPis with immune checkpoint inhibitors in EOCs may boost tumor neoantigen generation, promoting an antitumor immune microenvironment. Furthermore, agents interfering with HR proficiency may foster antitumor immune response by increasing the release of damaged DNA from cancer cells, thus triggering the activation of the host’s innate immune system [56,57].

Underpinned by this evidence, several clinical trials assessing the safety and efficacy of immune-checkpoint blockade in EOC are currently ongoing, testing anti-PD-1/PD-L1 and/or anti-CTLA-4 agents alone or, more frequently, in combination with conventional platinum-based chemotherapy or targeted therapies (Table 2).

Specifically, as far as the neoadjuvant and post-operative settings are concerned, the phase 3 IMagyn050 trial (NCT03038100) aims at investigating the safety and efficacy of Atezolizumab, a fully humanized monoclonal antibody that targets PD-L1, versus placebo when administered in combination with Paclitaxel, Carboplatin and Bevacizumab (i.e., anti-VEGF humanized monoclonal antibody) in both women harboring stage III or IV EOC eligible for neoadjuvant therapy followed by interval debulking surgery and patients undergoing primary cytoreductive surgery with macroscopical residual disease postoperatively who necessitate of post-surgical systemic treatment.

Shifting focus on the first-line setting, the ATHENA clinical trial (NCT03522246) is evaluating the efficacy of maintenance therapy with the PARPi Rucaparib combined with the fully human anti-PD-1 monoclonal antibody Nivolumab in newly-diagnosed ovarian cancer patients who showed either complete or partial response to front-line platinum-based chemotherapy. In the same treatment setting, the MITO25 clinical trial (NCT03462212) is evaluating the potential synergism deriving from the combination of anti-angiogenic agents with PARPis by assessing the activity of three different drug regimens sharing a common platinum-based chemotherapy backbone. Specifically, treatment-naïve, advanced, or metastatic OC patients are randomly assigned to receive platinum-based chemotherapy followed by Rucaparib maintenance therapy versus platinum-based chemotherapy plus Bevacizumab in combination and as maintenance therapy versus platinum-based chemotherapy plus Bevacizumab followed by Bevacizumab plus Rucaparib maintenance therapy.

Most ongoing clinical trials are exploring the effectiveness of immune-checkpoint inhibitors in the recurrence scenario and their potential exploitation in late therapeutic lines. In this light, a phase 1/2 clinical trial (KEYNOTE-162; NCT02657889) has been designed to evaluate the safety and efficacy of combination treatment with the PARPi Niraparib and Pembrolizumab, a humanized monoclonal antibody targeting the PD-1 receptor, in recurrent EOCs. In another study (NCT02873962), Nivolumab is being tested in association with Bevacizumab alone or with Bevacizumab plus Rucaparib both in platinum-resistant and in platinum-sensitive relapsed EOC; the phase 3 ATALANTE trial (NCT02891824) is instead enrolling women with platinum-sensitive recurrent EOC to be randomly assigned to receive Atezolizumab in combination with Bevacizumab and platinum-based chemotherapy or placebo plus Bevacizumab and a platinoid-containing schedule. The aforementioned studies are ongoing and results are eagerly awaited.

Promising insights derive from the phase 1b JAVELIN clinical trial (NCT01772004) which demonstrated the acceptable safety profile and clinical activity of the anti-PD-L1 fully humanized antibody Avelumab in heavily pre-treated patients with EOC; interestingly, patients exhibiting PD-L1-positive tumors displayed a higher overall response rate with respect to women diagnosed with PD-L1-negative EOCs.

## 3. Mechanisms of Primary and Acquired Resistance to Platinum Salts and PARP Inhibitors in HGSOC

**Figure 3:**
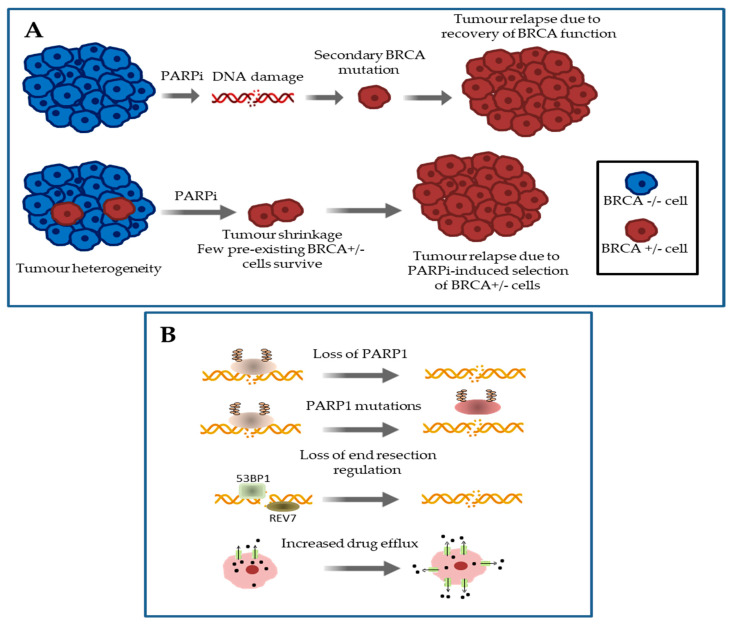
Mechanisms of resistance to PARPis. (**A**) BRCA-dependent mechanisms: (upper part) appearance of secondary “revertant” BRCA mutations (favored by increased DNA mutation rate) that restore the open reading frame and allow the synthesis of a functional BRCA protein; (lower part) PARPi-induced selection of pre-existing cells with “revertant” BRCA mutations. (**B**) BRCA-independent mechanisms: (from top to bottom) loss of PARP1 expression (often due to epigenetic mechanisms); appearance of PARP1 mutations altering PARP1 trapping; inactivation of the DNA repair proteins 53BP1 or REV7, resulting in the restoration of homologous recombination repair; increased expression of multidrug resistance proteins.

A further crucial chapter in HGSOC natural history is represented by the emergence of resistance to platinum-based chemotherapy and PARP inhibitors [26,58]. Indeed, the widespread use of germline and tumor DNA sequencing and the approval of PARP inhibitors in everyday clinical practice have led to a greater number of patients harboring BRCA 1/2 mutated ovarian tumors being treated with platinum agents and/or PARPis, on one hand, and to the problem of acquired resistance to these therapeutic regimens, on the other hand. Huge efforts in clinical research have been made trying to identify molecular mechanisms underlying resistance in homologous recombination deficient HGSOCs and different alterations have been reported (Figure 3A,B) [59,60,61,62,63].

Among resistance mechanisms, reversion mutations in BRCA1 or BRCA2 genes that partly restore wild-type protein function constitute the most common key mechanism leading to platinum compounds and PARPis resistance in HGSOCs (Figure 3A). Specifically, reversion mutations are represented by secondary somatic mutations, i.e., base substitutions or, more often, insertions/deletions (indels), in a mutant BRCA 1/2 allele which restore the open reading frame of the gene, heading to the expression of a partly functional protein product [26]. In brief, reversion mutations switch neoplastic cells from an HR-deficient to an HR-proficient phenotype and promote drug resistance by enabling DNA-damage repair induced by PARPis and/or platinum-based chemotherapy, undermining the basis of synthetic lethality and ultimately leading to tumor cells survival [3,26,58].

It has been demonstrated that the restoration of BRCA 1/2 functionality can occur in the setting of either germline or somatic BRCA 1/2 mutations and that different tumor sites within one patient may harbor distinct reversion mutations, highlighting the great spatial and temporal intratumoral heterogeneity which basically characterizes HGSOCs [3]. This striking heterogeneity draws attention to the importance of having a real-time “global picture” of molecular characteristics typifying each individual HGSOC, in order to tailor the best therapeutic intervention for every single patient. In this scenario, analysis of circulating cell-free DNA (cfDNA) may identify multiple reversion mutations simultaneously, at the time of or prior to clinically detectable resistance to platinum compounds or PARPis. Interestingly, in order to evaluate the prevalence of BRCA reversion mutations in HGSOCs, Lin et al. [64] conducted targeted next-generation sequencing (NGS) of circulating cell-free DNA extracted from pre-treatment and post-progression plasma in patients with deleterious germline or somatic BRCA mutations treated with the PARP inhibitor Rucaparib. BRCA reversion mutations were identified in pre-treatment cfDNA from 18% of platinum-refractory and 13% of platinum-resistant cancers, compared with only 2% of platinum-sensitive cancers. Of note, patients carrying BRCA-mutant cancers without detectable BRCA reversion mutations in pre-treatment cfDNA analysis showed significantly longer progression-free survival after treatment with Rucaparib when compared to those with pre-treatment reversion mutations (median PFS 9.0 vs. 1.8 months; HR 0.12; *p* < 0.0001). Furthermore, since this positive trend was also unveiled within the platinum-resistant and platinum-refractory BRCA-mutant cohorts (median PFS 7.3 vs. 1.7 months; HR 0.16; *p* < 0.0001), this finding gave some appealing insights on the potentially beneficial role of PARPis in women whose cancers retain BRCA mutations after progressing within 6 months after the completion or during their last platinum-based chemotherapy. As far as acquired resistance to Rucaparib is concerned, by analyzing plasma cfDNA at time when progression to Rucaparib occurred, Lin and collaborators detected eight extra patients harboring BRCA reversion mutations not mapped in pre-treatment cfDNA. Captivatingly, in four of the eight patients with acquired BRCA reversion mutations, the reversion mutations were detected in plasma samples collected prior to clinical progression (assessed by RECIST (Response Evaluation Criteria In Solid Tumors) criteria) and, specifically, at a median of 3.4 months (range 0.7–8.3 months) before progression. By contrast, the remaining four patients had BRCA reversion mutations detected in plasma samples only at the time of radiologic progression. Taken together, these results underline how the detection of BRCA reversion mutations by cfDNA analysis is associated with forthcoming or concurrent cancer progression and may warrant a change in the adopted therapeutic approach: HGSOCs which harbor a BRCA reversion mutation and progress during one single-PARPi therapy should not be treated with another single-agent PARPi-based strategy.

Furthermore, only a little subgroup within patients with platinum-resistant and platinum-refractory HGSOCs revealed BRCA reversion mutations in pre-treatment and post-progression cfDNA, pointing at the presence of other mechanisms involved in primary and acquired resistance to platinum agents and PARPis. Among these, reversion mutations in other tumor suppressor genes associated with the DNA repair machinery, such as PALB2, RAD51C, and RAD51D, have been described [64].

As a consequence, in order to better predict sensitivity to platinum-based chemotherapy and PARPis, assays aimed at distinguishing cancers with ongoing genomic instability from those with just a history of genomic instability followed by functional restoration of DNA repair defects are eagerly awaited [3]. Indeed, since the purpose of next generation clinical trials consists in evaluating the effectiveness of different therapies after PARPis progression, patients should be stratified for the presence of reversion mutations in tumor tissue and/or cfDNA at the time of trial entry. In the near future, cfDNA analysis could effectively support oncologists in the clinical management of HGSOCs, unveiling the probability of tumor response or the risk of tumor refractoriness/progression in the setting of a drug regimen consisting of platinum-based compounds or PARPis [64]. In this scenario, it could be employed to enhance and refine the predictive power of the well-known “platinum-free interval” (PFI), which, regrettably, constitutes the only predictive marker of response currently used to guide drug selection in relapsed HGSOCs and which is not effective in discriminating, among platinum-resistant and platinum-refractory patients, those who would benefit from a PARP inhibitor-based schedule despite their clinical behavior (assessed by PFI) following a platinum-based regimen [26,58,64].

Other processes involved in DNA repair pathways that mediate resistance to PARPis but are not related to BRCA genes are represented by the decreased expression of PARP enzymes [61], the onset of PARP1 mutations altering its trapping on DNA [61], the inactivation of the DNA repair proteins 53BP1 (i.e., tumor protein p53 binding protein 1) [65,66,67] or REV7 [68,69] and the increased expression of ATP-binding cassette transporters (i.e., the p-glycoprotein efflux pump, also known as multidrug resistance protein 1) [62] (Figure 3B).

In particular, 53BP1 is a chromatin binding protein that regulates DNA repair by inhibiting DNA end resection and homologous recombination by binding to double strand breaks and promoting non-homologous end joining [65]. It has been shown that loss of 53BP1 in BRCA1 null cells promotes homologous recombination, conferring PARPis resistance [65,66,67]. REV7 has recently been identified as a component of the shieldin complex, a downstream effector of 53BP1 in DNA double strand break repair [68]. Loss of REV7 re-establishes end resection of DSBs in BRCA1-deficient cells, leading to HR restoration and PARP inhibitor resistance [69].

More recently, focusing on the PARP-trapping mechanism of action of PARPis, researchers have pointed at the restoration of replication fork stability as another emerging way for cancer cells to withstand PARP inhibition; indeed, cytotoxic death deriving from PARPis-induced stalled replication forks may be bypassed by parallel compensatory pathways restoring and restarting the DNA-repair machinery [63].

## 4. Issues with the Management of HR Proficiency in HGSOC

The ENGOT-OV16/NOVA trial results [39] underscored the efficacy gap of Niraparib maintenance therapy between patients harboring HRD-positive HGSOCs and their HRD-negative counterpart. Indeed, even though Niraparib significantly reduced the risk for disease progression in the HRD-negative patient subpopulation, this beneficial effect was considerably lower when compared with the magnitude of PFS improvement achieved in the HRD-positive subgroup. This evidence emphasizes both the marginal impact of PARPis in the treatment of HRD-negative OC patients and the need for new therapeutic strategies targeting HR-proficient ovarian cancers.

The detection of BRCA1/2 reversion mutations during the natural history of HGSOC highlights how the loss of BRCA1/2 function and its associated DNA-repair defect seem to be required only for initiation of tumorigenesis and not needed for maintenance of the cancer phenotype. As a consequence, treating BRCA1/2-deficient HGSOCs by trying to restore BRCA1/2 function could represent an ineffective strategy and might render these cancers even more fit. According to Rojas and collaborators [7], this phenomenon can be referred to as “tumor-suppressor tolerance”, in order to place it in contrast to “oncogene addiction”. In this landscape, it is quite straightforward to understand how challenging the purpose to find ways to overcome de novo and acquired homologous-recombination proficiency is.

**Figure 4:**
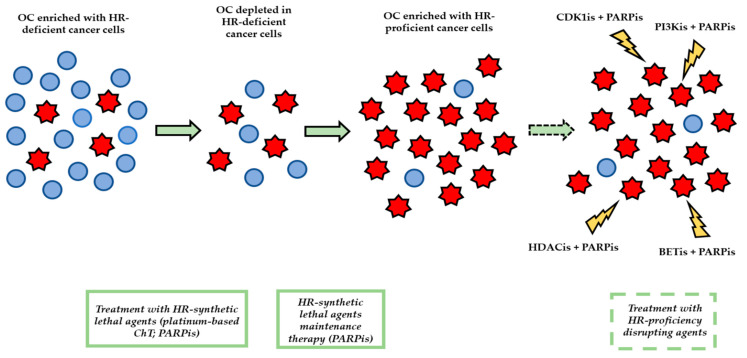
Ovarian cancer (OC) tumor evolution under treatment with HR-synthetic lethal agents and new insights into novel therapeutic approaches aiming at disrupting HR proficiency in OC. Legend. Blue circle: HR-deficient ovarian cancer cell; Red star: HR-proficient ovarian cancer cell; OC: Ovarian Cancer; HR: Homologous Recombination; ChT: Chemotherapy; PARPis: Poly (ADP Ribose) Polymerase Inhibitors; CDK1is: Cyclin-Dependent Kinase 1 inhibitors; PI3Kis: PhosphoInositide 3-Kinase inhibitors; BETis: Bromodomain and Extraterminal Domain inhibitors; HDACis: Histone DeACetylase inhibitors.

A fascinating and pioneering strategy consists in an attempt to convert HR-proficient tumors into HR-deficient phenotypes by means of drugs which aim at disrupting HR pathway through different mechanisms (Figure 4). The objective of this paradigm is to combine platinum compounds and PARPis with agents that functionally abrogate HR, in order to confer a “BRCAness” phenotype to HR-proficient tumors, enhancing the therapeutic activity of platinoids and PARPis beyond HR-deficient HGSOCs.

In this setting, several approaches meant to selectively interfere with DNA HR-repair and to sensitize cancer cells to platinum or PARPis have been evaluated, both preclinically and in early clinical trials.

Johnson et al. [70] demonstrated how cyclin-dependent kinase 1 (CDK1) is deeply implicated in HR, since CDK1-mediated BRCA1 phosphorylation constitutes an essential step for HR machinery effector scaffolding upon DNA DSBs: by combining CDK1 and PARP inhibition, they obtained reduced cell colony formation, disrupted human tumor xenograft growth and tumor regression leading to improved survival in a mouse model of BRCA WT lung adenocarcinoma. Therefore, targeting CDK1 activity could impair the HR DNA repair machinery and render transformed cells more prone to the cytotoxic activity of PARPis. On the wave of the same speculative principle, other intriguing studies have been performed over time. Ibrahim and collaborators [71] demonstrated that phosphoinositide 3-kinase (PI3K) inhibition could be exploited to induce HR deficiency and sensitization to PARPis in BRCA-proficient triple-negative breast cancers, since PI3K closely interacts with the HR complex in order to stabilize and preserve DSB repair, while other researchers focused their attention on exploring the role played by epigenetic mechanisms in HR efficiency. In particular, Vorinostat [72] and Panobinostat [73], two histone deacetylase inhibitors (HDACis), were shown to downregulate genes implicated in HR DNA repair, such as cyclin E, E2F1, and BRCA1, in HR-proficient ovarian cancer cells, leading to synergistic enhancement of cytotoxicity when these compounds were combined with the PARPi Olaparib. Of note, the cytotoxic effect of the combination between Panobinostat and Olaparib was striking chiefly in cyclin E-overexpressing HR-proficient ovarian tumor cells. This finding could be explained by the intrinsic reliance of cyclin E-overexpressing HGSOCs on high levels of HR proficiency; indeed, since amplified cyclin E is a well-known oncogenic driver of unchecked replication which leads to replicative stress and great genomic instability, cyclin E-overexpressing ovarian tumors are strictly dependent on robust HR DNA repair mechanisms for their survival [73].

As far as epigenetic mechanisms are concerned, another molecular player which seems deeply involved in HGSOC homologous recombination DNA repair is represented by the bromodomain and extraterminal domain (BET) protein BRD4. BRD4, along with BRD2 and BRD3, acts as an epigenetic reader in order to regulate gene transcription; particularly, since BET target genes’ products actively participate in key cellular processes involving cell cycle control, DNA repair, cell growth, cancer and inflammation, small molecule BET inhibitors (BETis) have drawn researchers’ attention lately [74]. Of note, according to The Cancer Genome Atlas database, BRD4 gene is amplified in approximately 10% of HGSOCs, laying the groundwork for BETis to represent a potentially effective therapeutic strategy in this subtype of tumors with poor clinical outcome. Specifically, by both knocking down and inhibiting BRD4 via the novel BETi INCB054329, Wilson et al. [75] observed a substantial reduction in the expression and function of HR components, in particular BRCA1 and RAD51, both in cultured ovarian cell lines and in patient-derived xenografts; furthermore, they noticed enhanced tumor cell growth inhibition, DNA damage generation, and apoptosis when BRD4 pathway disruption was associated with treatment with Olaparib.

Along with the improvements made in the knowledge of mechanisms underlying epigenetic regulation of gene expression, other molecular targets have been studied and exploited in order to turn HR-proficient HGSOCs into HR-deficient ones. Of note, attractive molecular targets are constituted by: the heat shock protein 90 (HSP90), which is an ATP-dependent molecular chaperone mediating the maturation, stability and activation of several hundreds of different proteins, including cell cycle regulators, such as CDK1, and key proteins essential for DNA repair, such as BRCA1, BRCA2, and RAD51 [76]; cyclin D1, a component of the cell cycle machinery which is involved in HR-mediated DNA repair and is overexpressed in 14–89% of ovarian cancer cases, resulting associated with a poorer prognosis [77]; vascular endothelial growth factor receptor 3 (VEGFR3), in which inhibition resulted in cell cycle arrest, decrease of both BRCA1 and BRCA2 expression, and significant increase of chemosensitivity in resistant ovarian cell lines in which a BRCA2 mutation had reverted to wild-type [78].

## 5. DNA Mismatch Repair Deficiency in EOC and Deriving Clinical Implications

Focusing on the other DNA repair mechanism, i.e., DNA mismatch repair (MMR), it is necessary to highlight how this field of research has been poorly systematically investigated in the landscape of EOCs [8,9,79].

MMR is involved in the correction of DNA base-base mismatches and insertion/deletion mismatch repairs generated during DNA replication, and its deficiency is associated with an increased risk of developing several types of cancer, constituting the most common cause of hereditary ovarian cancer after BRCA1/2 mutations [79,80,81]. Even though germline MMR gene mutations occur in only 2% of ovarian carcinomas, the incidence rate of MMR deficiency rises to 29% when considering expression loss of one of the seven MMR main genes (i.e., MSH2, MSH3, MSH6, MLH1, MLH3, PMS1, and PMS2). Interestingly, both mutational and expression data point at non-serous ovarian carcinomas as the histologies that more frequently display MMR deficiency [8]. Unfortunately, findings concerning the prognostic landscape in MMR-deficient ovarian cancers are highly controversial. Whereas numerous studies have explored survival in MMR-deficient colorectal cancers, survival and treatment outcomes in MMR-defective ovarian cancers are still hugely under-investigated. As far as colorectal cancer is concerned, after reviewing 32 eligible studies which stratified survival in colorectal cancer patients by microsatellite instability (MSI) status, Popat et al. [82] confirmed that MSI-high status is associated with enhanced survival. Moreover, Radman and Wagner [83] reported the relationship intervening between MSI-related genetic instability and compromised cancer progression, suggesting its implication in improving colorectal cancer patient survival. By contrast, since only few studies with diverging or inconclusive results have been investigating the role played by DNA MMR deficiency in EOCs, its implications in both EOC prognosis and clinical response to different therapeutic regimes remain uncovered [84,85,86,87,88,89,90,91,92]. However, despite the scarce scientific evidence concerning MMR-deficient EOC, an appealing insight into this undiscovered and niche landscape is represented by a distinctive subset of clear cell ovarian carcinomas (CCOC) characterized by microsatellite instability. Howitt [93] described for the first time an epidemiologically rare but clinically relevant cohort composed by MMR deficient CCOCs, highlighting how they were associated with a significantly higher number of TILs and PD-1-positive TILs with respect to their microsatellite-stable counterpart and HGSOC; furthermore, MSI CCOCs homogenously expressed PD-L1 in the tumor cells and/or in the intraepithelial or peritumoral immune cells. These unique immunogenic biological features pointed at the MSI CCOC subset as a favored candidate for immune-checkpoint blockade, offering this uncommon, standard-chemotherapy resistant histologic subtype a promising effective therapeutic alternative [93].

## 6. Conclusions and Future Perspectives

Despite noteworthy improvements achieved in surgical and medical treatments, high-grade serous ovarian cancer still represents the deadliest gynecologic malignancy, essentially due to the high tendency to relapse and develop resistance to platinum-based chemotherapy during its natural history. Huge preclinical and clinical efforts have been made to cope with these issues, particularly focusing on DNA repair deficiency and strategies to exploit this distinctive feature, which is regarded as “the Achille’s heel” of HGSOC.

Two DNA repair mechanisms are mainly investigated and targeted in cancers, i.e., DNA homologous recombination and DNA mismatch repair pathways. As far as MMR pathway is concerned, this field of research remains poorly systematically assessed in epithelial ovarian cancer and evidence concerning survival and prognosis features and potential treatment approaches in microsatellite-instable EOCs is actually controversial and inconclusive. On the other hand, DNA HR deficiency and its clinical implications have been deeply explored in HGSOCs, leading to both a greater understanding of biological and clinical characteristics of HRD-positive ovarian carcinomas and the introduction of novel therapeutic drugs acting as synthetic lethal to HR pathway alterations. Germline and somatic BRCA 1/2 mutations represent the most frequent alterations interfering with HR in HGSOCs; striking improvements in BRCA 1/2-mutated HGSOC natural history have been reached with PARP inhibitors in clinical practice. Nonetheless, the widespread use of PARPis has determined the emergence of resistance to this innovative therapeutic approach, principally related to reversion mutations restoring BRCA 1/2 activity. As a consequence, research attention has been driven to both assess new targets able to revert a HR-proficient tumor phenotype to a HR-deficient one and investigate potential antitumor activity of immune-checkpoint inhibitors in TILs-enriched EOCs. Several promising preclinical and clinical trials are ongoing and their results are eagerly awaited. However, despite increasing knowledge about HGSOC biological and molecular landscapes, a “grey zone” concerning HGSOC biological behavior and implications of intratumoral and spatial/temporal heterogeneity still remains uncovered. Clinical trials relying on firm scientific bases and solid translational research are keenly needed in order to address these unmet preclinical and clinical answers. Future insights would possibly derive from next-generation sequencing analyses conducted on circulating cell-free DNA.

## Figures and Tables

**Table 1:** *FDA* and EMA approvals for Poly (ADP-ribose) Polymerase (PARP) inhibitor (PARPis) Monotherapy in ovarian cancer (OC).

Drug	Maintenance Therapy after Response To First-Line Platinum-Based ChT	Maintenance Therapy after Response To Platinum-Based ChT in Recurrence Setting	Monotherapy in Recurrence Setting
**OLAPARIB** 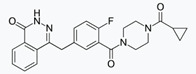	Germline or somatic BRCA1/2-mutated HGSOC	Germline or somatic BRCA1/2-mutated PS recurrent HGSOC *BRCA1/2-wild-type PS recurrent HGSOC*	*Germline BRCA1/2-mutated recurrent HGSOC, following 3 or more prior ChT lines*
**RUCAPARIB** 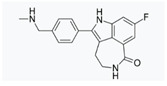	-	PS recurrent HGSOC	Germline or somatic BRCA1/2-mutated PS recurrent HGSOC, following 2 or more prior platinum lines (pts not candidate to further platinum) *Germline or somatic BRCA1/2-mutated recurrent HGSOC, following 2 or more prior ChT lines*
**NIRAPARIB** 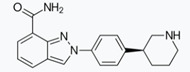	*BRCA1/2-mutated and BRCA1/2 WT HGSOC*	PS recurrent HGSOC	-

FDA: Food and Drug Administration; EMA: European Medicines Agency; ChT: Chemotherapy; HGSOC: High-grade Serous Ovarian Cancer; PS: Platinum Sensitive; in black: approvals by both FDA and EMA; *italics: approvals by FDA only*; underlined: approvals by EMA only.

**Table 2:** Main clinical trials with immune checkpoint inhibitors in epithelial ovarian cancers.

**Trial Name/Identifier**	**Phase**	**Condition**	**Therapeutic Setting**	**Drug Regimen(s)**	**Primary Endpoint**	**Status**
IMagyn050/NCT03038100	III	Stage III-IV EOC, FTC and PPT	Neoadjuvant/post-operative	Atezolizumab + Paclitaxel, Carboplatin and Bevacizumab vs. Placebo + Paclitaxel, Carboplatin and Bevacizumab	PFS and OS ITT/PDL-1+ population	Active, not recruiting
ATHENA/NCT03522246	III	Stage III-IV EOC, FTC and PPT	Maintenance after CR or PR to first line platinum-based ChT	Rucaparib + Nivolumab vs. Rucaparib + Placebo vs. Placebo + Nivolumab vs. Placebo + Placebo	PFS	Recruiting
MITO 25/NCT 03462212	I/II	Stage III-IV EOC, FTC and PPT	First line	Carboplatin, Paclitaxel + Rucaparib (only in maintenance) vs. Carboplatin, Paclitaxel + Bevacizumab (in combination and maintenance) vs. Carboplatin, Paclitaxel + Bevacizumab (in combination and maintenance) + Rucaparib (only in maintenance)	PFS and Safety	Recruiting
KEYNOTE-162/NCT02657889	I/II	Advanced and metastatic TNBC, EOC, FTC and PPT	First and subsequent lines	Niraparib + Pembrolizumab	ORR and Safety	Active, not recruiting
NCT02873962	II	Progressive or recurrent EOC, FTC and PPT	Second, third or fourth line	Nivolumab + Bevacizumab vs. Nivolumab + Bevacizumab and Rucaparib	ORR	Recruiting
ATALANTE/NCT02891824	III	Progressive or recurrent EOC, FTC and PPT	Second or third line	Atezolizumab + Bevacizumab and platinum-based ChT followed by Atezolizumab maintenance vs. Placebo + Bevacizumab and platinum-based ChT followed by Placebo maintenance	PFS	Active, not recruiting
JAVELIN/NCT01772004	I	Metastatic or locally advanced solid tumors	Progressive disease following last “standard-of-care” line of treatment	Avelumab	BOR and Safety	Completed
ANITA/NCT03598270	III	Progressive or recurrent EOC, FTC and PTT	Second or third line	Atezolizumab + platinum-based ChT followed by Atezolizumab and Niraparib maintenance vs. Placebo + platinum-based ChT followed by placebo and Niraparib maintenance	PFS	Recruiting
MEDIOLA/NCT02734004	I/II	Advanced solid tumors	Relapsed disease following “standard-of-care” treatment	Olaparib + MEDI4736 (Anti-PDL-1 Antibody)/Olaparib + MEDI4736 (Anti-PDL-1 Antibody) + Bevacizumab	DCR, ORR and Safety	Recruiting
MITO27/NCT03539328	II	Progressive or recurrent EOC, FTC and PPT	Second or third line	Pegylated liposomal Doxorubicin or weekly Paclitaxel or Gemcitabine (at Physician’s discretion) vs. Pegylated liposomal Doxorubicin + Pembrolizumab or weekly Paclitaxel + Pembrolizumab or Gemcitabine + Pembrolizumab (at Physician’s discretion)	OS	Not yet recruiting

EOC: Epithelial Ovarian Cancer; FTC: Fallopian Tube Cancer; PPT: Primary Peritoneal Tumor; TNBC: Triple-Negative Breast Cancer; ChT: Chemotherapy; PFS: Progression Free Survival; OS: Overall Survival; ORR: Objective Response Rate; BOR: Best Overall Response; DCR: Disease Control Rate; CR: Complete Response; PR: Partial Response; ITT: Intention-To-Treat; PDL-1+: Programmed Death Ligand1. For more detailed information, see www.clinicaltrials.gov.
